# Risk-Adaptive Cardio-Oncology Rehabilitation: A Narrative Review of Exercise Prescription, Multimodal Monitoring, and Implementation Pathways

**DOI:** 10.3390/healthcare14162596

**Published:** 2026-08-18

**Authors:** Min Luo, Xiangeng Hou, Yangguang Yu, Yingying Zheng, Xiang Xie

**Affiliations:** Department of Cardiology, First Affiliated Hospital of Xinjiang Medical University, Urumqi 830054, China; luomin9999@outlook.com (M.L.); hxg125@xjmu.edu.cn (X.H.); c1emasuka@gmail.com (Y.Y.)

**Keywords:** cardio-oncology rehabilitation, healthcare delivery, exercise prescription, FITT-VP, global longitudinal strain, telerehabilitation, cancer survivorship, implementation science

## Abstract

**Highlights:**

**What are the main findings?**

- This narrative review separates direct clinical evidence, guidance and synthesis, feasibility evidence, and author-derived conceptual proposals across exercise prescription, monitoring, and implementation.
- Direct clinical evidence supports selected fitness, functional, and intermediate outcomes; the proposed frequency, intensity, time, type, volume, and progression (FITT-VP)–based prescription and monitoring pathway is an author-derived, hypothesis-generating framework, not a validated algorithm.

**What are the implications of the main findings?**

- Cardio-oncology rehabilitation (CORE) may connect cardiovascular risk assessment with exercise-centered adult cancer care, but current evidence is concentrated in selected populations and intermediate outcomes.
- Therapy-specific safety, cardiovascular endpoints, sex and cancer-type generalizability, scalable delivery, equity, and cost-effectiveness require prospective evaluation.

**Abstract:**

**Background/Objectives**: Cardiovascular toxicity and pre-existing cardiovascular disease can affect cancer-treatment tolerance, functional recovery, and survivorship. This narrative review aimed to summarize current evidence and propose an author-derived risk-adaptive clinical framework for adult cardio-oncology rehabilitation (CORE), organized around exercise prescription, multimodal monitoring, and implementation. **Methods**: PubMed/MEDLINE and the Web of Science Core Collection were searched from database inception through 27 July 2026. Guidelines, systematic reviews, randomized and non-randomized clinical studies, feasibility studies, and selected mechanistic sources were prioritized according to their relevance to the three review domains. Evidence was classified as direct clinical, guidance/synthesis, feasibility/implementation, or mechanistic/conceptual; no PRISMA protocol, formal risk-of-bias assessment, or meta-analysis was undertaken. **Results**: Clinical trials support improvements in cardiorespiratory fitness, selected cardiovascular risk factors, and functional outcomes in some settings, but effects on cancer therapy-related cardiac dysfunction, cardiovascular events, and mortality remain uncertain. Direct evidence is dominated by breast-cancer cohorts and women, although mixed-cancer, lymphoma, and lung-cancer studies broaden the functional evidence base. An evidence-informed approach is to individualize aerobic and resistance exercise to treatment context, symptoms, functional capacity, and clinical stability, with reassessment linked to actionable findings; the proposed pathway remains conceptual and non-validated. Artificial intelligence, digital twins, omics, and monitoring-guided exercise-dose adjustment remain investigational. **Conclusions**: CORE is best regarded as exercise-centered care linked to guideline-based risk assessment and clinically indicated reassessment. Prospective studies should test therapy-specific timing, risk-stratified delivery, monitoring-guided dose adjustment, clinical endpoints, equity, and cost-effectiveness.

## 1. Introduction

Cardiovascular disease (CVD) is an important competing health risk during cancer treatment and survivorship, affecting treatment tolerance, functional recovery, and long-term follow-up [1,2]. Anthracyclines can cause dose-related myocardial injury; anti-human epidermal growth factor receptor 2 (HER2) therapy can impair cardiomyocyte survival signaling; thoracic radiotherapy can produce delayed vascular, valvular, pericardial, and myocardial disease; and immune checkpoint inhibitors (ICIs) can cause acute myocarditis [3,4,5,6,7,8,9,10,11,12,13,14,15,16]. These distinct injury patterns support cardiovascular risk assessment before treatment, surveillance during therapy when indicated, and timely rehabilitation referral once clinical stability and exercise safety have been established.

Cardiac rehabilitation is an exercise-centered intervention with established value in conventional cardiovascular disease. Cardio-oncology rehabilitation (CORE) adapts selected components to cancer care by linking individualized aerobic and resistance exercise with cardiovascular risk-factor management and coordinated oncology–cardiology review [1,17,18]. In an observational cohort of 442 patients with cancer and established cardiovascular disease, 361 completed a 12-week cardiac rehabilitation program and improved cardiorespiratory fitness; the survival association was observational and cannot establish causality [19]. Randomized CORE studies have subsequently reported setting-specific functional, risk-factor, and imaging outcomes, but no trial has established reductions in cardiovascular events or mortality [20,21,22,23,24].

Translation into routine care remains uneven. Reviews describe heterogeneity in program content and access barriers, while clinician surveys document variable knowledge and monitoring practice [25,26,27]. The ICOS-CORE working group characterizes the evidence base as heterogeneous and dominated by fitness or intermediate outcomes rather than cardiovascular events [28]. Accordingly, this review separates four evidence roles: direct clinical studies; guidelines and systematic syntheses; feasibility or implementation evidence; and mechanistic or conceptual literature. This distinction is maintained in the text and the clinical-evidence table.

For service design, a staged model is more defensible than an all-or-none referral rule. One patient may begin education and low-intensity activity while awaiting full assessment, whereas another may need supervised exercise from the outset. The descriptive, review, and early trial evidence supports early identification of barriers and cardiovascular risk but does not define a universal referral threshold or program duration [20,25,26,27,28]. The workflow used in this review is therefore an author-derived clinical synthesis that requires validation: treatment-related risk informs the initial setting, and longitudinal reassessment informs progression or review.

This review focuses on three clinically actionable domains: (1) risk-adaptive exercise prescription; (2) multimodal monitoring and clinically indicated reassessment; and (3) implementation pathways for integrating CORE into adult cancer care. Its novelty lies not in a new guideline or an efficacy claim. Rather, it attempts to provide an evidence-to-decision structure that identifies which statements are supported by direct clinical data, which are extrapolated from guidance or feasibility studies, and which are authors’ proposals requiring validation. This structure complements European Society of Cardiology (ESC) risk and surveillance guidance [7], the American Heart Association (AHA) CORE components and referral framework [18], and the International Cardio-Oncology Society (ICOS)-CORE research standards [28].

## 2. Materials and Methods

### 2.1. Review Design and Scope

A narrative design was chosen because the objective was to integrate heterogeneous evidence—guidelines, systematic reviews, trials, cohorts, feasibility studies, and mechanistic literature—into a clinically oriented framework rather than estimate one pooled treatment effect. The target population was adults during cancer treatment or survivorship. Pediatric exercise and surveillance literature [29], palliative-care rehabilitation, and detailed non-exercise supportive interventions were outside the synthesis. These boundaries maintain a focused synthesis of adult, exercise-centered CORE across the three prespecified domains; pediatric, palliative, and non-exercise supportive-care contexts have distinct goals, populations, and evidence bases that require separate evaluation. Psychological, sleep, nutritional, and cachexia literature was used only to define this boundary and was not treated as CORE efficacy evidence [30,31,32,33,34]. Older seminal sources were retained when needed to explain treatment toxicity or establish clinical context.

### 2.2. Search Strategy

PubMed/MEDLINE and the Web of Science Core Collection were searched from database inception through 27 July 2026. Search concepts combined terms for cancer and treatment exposure (cancer, survivor, anthracycline, anti-HER2, radiotherapy, immune checkpoint inhibitor, hematologic malignancy, transplantation), rehabilitation and exercise (cardiac rehabilitation, cardio-oncology rehabilitation, exercise training, aerobic, resistance, frequency, intensity, time, type, volume, and progression [FITT-VP], telerehabilitation), monitoring (global longitudinal strain, ejection fraction, troponin, natriuretic peptide, cardiac magnetic resonance, coronary calcium, cardiopulmonary exercise testing), and implementation (referral, hybrid care, digital health, equity, access, cost, artificial intelligence, digital twin, omics). Reference lists of major guidelines and reviews were hand searched for additional clinical studies.

### 2.3. Eligibility, Selection, and Resolution of Disagreements

Eligible sources addressed adults with cancer or cancer survivorship and at least one prespecified domain: exercise prescription, cardiovascular monitoring, or CORE implementation. Priority was given to sources that reported treatment context, exercise exposure or delivery, cardiovascular or functional outcomes, safety, adherence, or implementation measures. Pediatric-only, palliative-only, non-cancer, and purely nutritional or psychological intervention studies were excluded from the focused synthesis; preclinical sources were retained only for biological rationale. M.L. and X.H. independently screened titles and abstracts and assessed potentially eligible full texts. Disagreements regarding study selection were adjudicated by Y.Z. Because this was a narrative review, a PRISMA flow diagram and formal study-count denominator were not generated.

### 2.4. Evidence Prioritization and Data Presentation

Evidence sources were matched to the type of claim. Contemporary guidelines and consensus statements were prioritized for clinical recommendations and surveillance; systematic reviews and randomized trials for intervention effects; prospective and retrospective cohorts for associations and routine-care experience; feasibility and implementation studies for delivery; and mechanistic or conceptual literature only for biological rationale or future research. The clinical-evidence table summarizes the adult clinical studies that directly anchor the main conclusions on CORE exercise efficacy, safety, or delivery, together with the principal systematic review used to contextualize this clinical evidence base. Guidelines and consensus statements, other systematic syntheses, mechanistic studies, and general background sources were integrated in the narrative according to their evidence role because study-level participant counts and intervention fields are not consistently applicable to them. Monitoring studies that did not evaluate a rehabilitation intervention were discussed in the relevant monitoring sections rather than treated as CORE efficacy studies. For the sources tabulated in that table, the authors extracted study/year, cancer population and sex distribution, design, sample size, intervention or exposure, main outcomes, and the appropriate evidence interpretation. Positive, neutral, and null findings were retained to avoid presenting an efficacy-only account. No formal risk-of-bias tool or GRADE rating was applied; the table is descriptive and is not a PRISMA study inventory, formal risk-of-bias assessment, or GRADE evidence profile.

## 3. Risk Stratification and the Rationale for CORE

### 3.1. Treatment-Related Cardiovascular Risk

Table 1 summarizes treatment mechanisms, likely reversibility, and monitoring markers relevant to rehabilitation planning. Anthracycline-related cardiac dysfunction is dose- and risk-dependent, and clinical consensus statements emphasize prevention, screening, monitoring, and prompt management throughout oncological treatment [9,10,11]. For CORE planning, these clinical data justify considering cumulative exposure, baseline cardiac status, symptoms, and early change during therapy rather than using a single mechanism-based exercise rule.

Reviews describe anti-HER2-related dysfunction as often more reversible than anthracycline injury and link it to interruption of ErbB2 survival and stress-adaptation signaling [5,12]. Recovery is not universal. Exercise initiation and progression should therefore reflect current ventricular function, symptoms, concomitant anthracycline exposure, and treatment phase, rather than an assumption of complete reversibility.

Radiotherapy-induced cardiovascular injury has distinctive pathological features related to dose and cardiac exposure. In breast-cancer radiotherapy, mean heart dose has been associated with a dose-dependent increase in subsequent ischemic heart disease risk [13]. Radiation-induced heart disease includes microvascular endothelial injury, inflammation, accelerated atherosclerosis, myocardial fibrosis, valvular disease, and pericardial disease [14]. Modern techniques aim to reduce cardiac exposure, but late injury remains a surveillance consideration [6]. These data support treatment-specific assessment; they do not establish a rehabilitation intervention that prevents radiation-related events.

Anthracycline-associated cardiomyopathy and ICI myocarditis require different rehabilitation decisions. Observational data suggest that early recognition of anthracycline-related left ventricular dysfunction and prompt heart-failure therapy can improve the likelihood of recovery [15]. Once clinically stable, exercise may be added as an adjunct to guideline-directed oncology and cardiovascular care, with supervision and dose determined by symptoms, ventricular function, treatment phase, and functional capacity. By contrast, suspected or active ICI myocarditis requires urgent evaluation and exercise deferral; rehabilitation should be considered only after clinical stabilization and specialist clearance [7,16,35]. CORE does not replace treatment of either disorder.

### 3.2. Exercise-Related Protective Rationale

Exercise has biologically plausible effects on mitochondrial function, endothelial health, inflammation, antioxidant signaling, and functional reserve. Preclinical doxorubicin models and translational reviews support this rationale but cannot define a safe clinical dose or demonstrate prevention of cancer therapy-related cardiac dysfunction (CTRCD) [36,37]. Mechanistic evidence is therefore used here to explain why exercise merits study, not to prescribe a molecularly targeted intervention.

Clinical prescription should instead rely on measured capacity, treatment context, symptoms, and the intervention actually tested. In breast cancer, randomized trials during anthracycline or anti-HER2 therapy have produced mixed or null findings for left ventricular ejection fraction (LVEF), global longitudinal strain (GLS), and biomarkers despite selected risk-factor or safety signals [20,21,23]. In lung-cancer survivors with reduced cardiorespiratory fitness, aerobic and combined training improved peak oxygen uptake compared with stretching, whereas resistance training alone did not; the trial also reported modality-specific differences in exercise tolerability [38]. These findings support explicit reporting of exercise type, intensity, duration, adherence, and population rather than assuming a uniform dose–response relationship across cancers.

Across the clinical literature, cardiorespiratory fitness, fatigue, function, risk factors, imaging, and biomarkers are reported more often than cardiovascular events or mortality [39,40]. Hypertension, dyslipidemia, diabetes, obesity, smoking, cancer cachexia, anemia, infection, and treatment toxicity can all alter exercise tolerance and should be assessed before dose progression [7,41,42]. These factors determine whether exercise can start, the appropriate setting, and when reassessment is needed; they do not create a universal referral threshold.

Risk-factor management is part of CORE rather than a separate secondary objective. Blood pressure, glucose, lipids, smoking, and body composition should be assessed alongside exercise tolerance, and abnormalities managed without delaying necessary cancer therapy. This combined assessment also helps distinguish a training limitation from uncontrolled comorbidity, treatment toxicity, anemia, infection, or another cause of reduced tolerance. Because combined rehabilitation has not been shown to prevent hard cardiovascular outcomes, near-term goals are safer participation, preserved function, and earlier recognition of deterioration.

Accordingly, Section 4 emphasizes clinically tested exercise interventions and prescription variables. Mechanistic sources provide context only, feasibility studies address delivery, and the authors’ proposed decision aids are labeled separately.

### 3.3. From Risk Stratification to CORE

**Figure 1:**
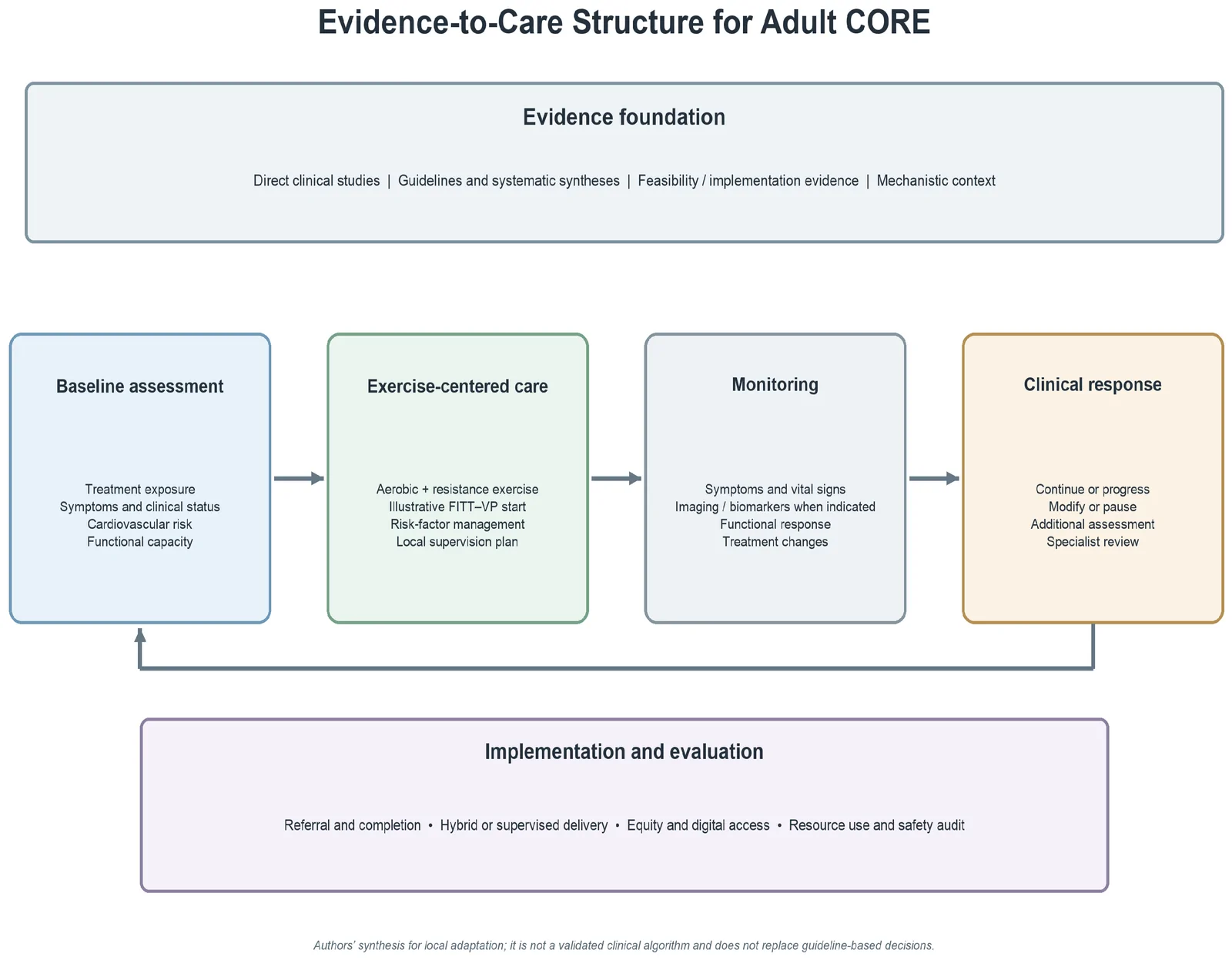
Evidence-to-care structure for adult CORE. Direct clinical studies, guidance and synthesis, feasibility or implementation evidence, and mechanistic context are distinguished before translation into assessment, exercise-centered care, monitoring, and clinical reassessment. This synthesis by the authors is conceptual, non-validated, and intended for local adaptation rather than replacement of guideline-based decision-making. CORE, cardio-oncology rehabilitation; FITT-VP, frequency, intensity, time, type, volume, and progression.

CORE reviews describe an exercise-centered model linking oncology, cardiology, and rehabilitation [1,17]. Figure 1 is the authors’ evidence-to-care synthesis: it places direct clinical evidence, guidance, feasibility data, and mechanistic context upstream of baseline assessment, exercise prescription, monitoring, and reassessment. It is intended to guide local pathway design, not to replace guideline-based decisions or imply that every patient requires every test.

Baseline assessment should integrate treatment history, cardiovascular risk factors, symptoms, electrocardiography, echocardiography, and biomarkers when clinically indicated [7]. ECG abnormalities contribute to toxicity assessment but are only one part of the evaluation [43]. Risk category then informs whether exercise can begin independently, requires supervised initiation, or should be deferred for cardiology review. Aerobic and resistance dose, progression, and monitoring frequency should account for active treatment, cytopenias, infection risk, functional capacity, and patient preference [44]. A baseline decision should record both the reason for the selected setting and the findings that would trigger reassessment; otherwise, risk categorization remains descriptive rather than actionable.

A systematic review in older cancer patients supports exercise-related functional outcomes but does not establish that CORE improves treatment tolerance or hard cardiovascular endpoints [45]. A review of cardiac rehabilitation research in cancer survivors also identifies limitations in study quality and standardization [40]. Effects on treatment completion, CTRCD prevention, survival, and cardiovascular events therefore remain uncertain. This distinction underpins the prescription approach used below.

## 4. Exercise-Centered CORE and Cardiovascular Risk Management

This first core domain summarizes direct exercise evidence and then separates the authors’ illustrative starting considerations from established clinical guidance.

### 4.1. Risk-Adaptive Exercise Prescription

Aerobic and resistance exercise are the central interventions considered in CORE, but the evidence is population- and outcome-specific. Systematic reviews in breast cancer primarily address cardiorespiratory fitness, cardiovascular risk factors, and intermediate cardiac measures [46,47,48]. Consensus exercise guidance supports individualization according to symptoms, function, treatment, comorbidity, and prior activity [49]. These sources support a structured prescription process; they do not validate the risk strata or monitoring triggers proposed in this review.

Randomized evidence is mixed. TITAN found no between-group benefit for LVEF or cardiac biomarkers after a 52-week cardiac-rehabilitation model, although total and low-density lipoprotein cholesterol improved [20]. ONCORE reported no CTRCD events, an attenuated LVEF decline, no GLS or biomarker difference, and no exercise-related adverse events in 122 women receiving potentially cardiotoxic breast-cancer therapy [21]. A separate anthracycline trial in breast cancer and lymphoma found no superiority of supervised exercise for GLS, biomarkers, or peak oxygen uptake [23]. A smaller 10-week CR trial in 29 women with subclinical cardiotoxicity during doxorubicin or trastuzumab treatment improved peak oxygen uptake relative to usual care but showed no between-group changes in GLS or high-sensitivity troponin [24]. These trials do not establish prevention of CTRCD.

Functional results also vary by population and delivery. In high-cardiovascular risk cancer survivors, supervised CORE improved peak oxygen uptake and selected risk factors compared with community-based exercise [22]. In lung-cancer survivors, aerobic and combined exercise improved peak oxygen uptake compared with stretching, whereas resistance training alone did not [38]. In lymphoma survivors, telehealth-supported home exercise produced short-term fitness and functional improvements comparable with center-based exercise, with high adherence and lower provider cost, but the trial was not designed for cardiovascular events [50].

The evidence base is therefore broader than breast cancer but remains uneven. Breast-cancer trials provide the most direct data during anthracycline or anti-HER2 therapy [20,21,23,46]. Lung-cancer studies inform modality-specific fitness and postoperative or survivorship rehabilitation rather than cardiotoxicity monitoring [38]. Lymphoma studies inform feasibility and delivery [50,51]; colorectal-cancer evidence chiefly addresses fatigue and function [52]; and prostate-cancer evidence is mainly functional and metabolic [53]. These differences should be preserved rather than combined into a single CORE efficacy claim.

The clinical-evidence table shows that most programs combined aerobic and resistance exercise two or three times weekly for 8–16 weeks, with reported session durations of 30–90 min; TITAN lasted 52 weeks and ONCORE a mean of 5.8 months. Aerobic intensity ranged from 50 to 85% of heart-rate reserve or 60–85% of maximal heart rate to intervals exceeding 95% of peak oxygen uptake, while reported resistance loads were approximately 40–85% of one-repetition maximum or maximal strength [20,21,22,23,24,38,50,51]. These heterogeneous trials do not define one optimal program. For a clinically stable adult, a cautious starting approach is individualized aerobic plus resistance exercise two to three times weekly at tolerable low-to-moderate intensity and duration, progressed according to symptoms, capacity, treatment, comorbidity, and supervision needs [49].

Table 2 presents abbreviated, illustrative starting considerations for four clinical contexts. It uses cautious language because no cited trial validated these categories, monitoring schedules, or escalation rules. Local protocols should be co-developed by oncology, cardio-oncology, and rehabilitation teams and adapted to staffing, diagnostic capacity, treatment mix, and emergency pathways.

### 4.2. Risk-Factor and Pharmacological Management

Risk-factor and pharmacological management should complement exercise prescription. Blood pressure, glucose, lipids, smoking, body composition, and medication interactions should be reviewed at baseline and follow-up. Cardioprotective medication can be considered for selected high-risk patients, but indication, timing, dose, interaction, and adverse effects require specialist review [54]. Guidelines define roles for GLS and cardiac magnetic resonance (CMR) in evaluating suspected dysfunction; evidence that either should automatically prompt a change in exercise dose is limited [7,55,56]. Suspected ICI myocarditis or other acute cardiovascular toxicity requires urgent clinical evaluation and exercise deferral until stable [7,16,35]. Medication review is also relevant when exercise symptoms could reflect hypotension, bradycardia, arrhythmia, volume status, or drug interaction. CORE staff should identify and communicate these findings but should not independently initiate or withdraw cardioprotective therapy.

For anthracycline exposure, cardioprotection evidence favors individualized rather than routine preventive medication [57]. A small randomized open-label trial evaluated ivabradine for anthracycline cardioprotection, but its findings are preliminary and do not justify routine use [58]. Medication decisions remain within oncology–cardiology care, with CORE contributing structured follow-up and functional assessment.

## 5. Multimodal Monitoring-Guided Adjustment

This second core domain distinguishes the established diagnostic roles of cardiovascular tests from the unproven proposition that test changes should automatically alter exercise dose.

### 5.1. GLS for Early Functional Change

GLS measures longitudinal myocardial deformation and can identify subclinical functional change before an overt LVEF decline. A meta-analysis evaluated its prognostic value for chemotherapy-induced cardiotoxicity [56]. For CORE, this supports cautious trend interpretation alongside treatment exposure, symptoms, LVEF, biomarkers, and exercise capacity, rather than use of a single cutoff. Serial GLS has been proposed for integrated monitoring, but GLS-guided exercise adjustment has not been shown to improve clinical outcomes [55].

### 5.2. Clinical Interpretation of GLS

Meta-analytic evidence supports a prognostic association between GLS and subsequent CTRCD, not a universal exercise rule [56]. A relative GLS decline greater than 15% is used in contemporary cardio-oncology guidance as a signal for clinical interpretation rather than an automatic treatment decision [7]. Serial values should be considered with LVEF, symptoms, biomarkers, exposure, image quality, and loading conditions. Exercise-associated GLS changes remain hypothesis-generating [55].

Automated GLS measurement can reduce some operator dependence, but artificial intelligence (AI)-based assessment remains investigational in cardio-oncology. Any AI-enabled selection or automated-measurement workflow should be treated as clinical decision support and evaluated for external validity, workflow integration, safety monitoring, and prospective clinical utility before it informs CORE prescriptions [59,60].

### 5.3. Complementary Assessment Tools

Cardiac magnetic resonance (CMR) provides tissue characterization when edema, fibrosis, or suspected myocarditis requires clarification within a clinical cardio-oncology assessment [7]. High-sensitivity troponin and natriuretic peptides indicate myocardial injury or hemodynamic stress but must be interpreted with symptoms, treatment timing, imaging, renal function, and other clinical factors [7]. Cardiopulmonary exercise testing (CPET) directly measures cardiorespiratory fitness and characterizes integrated exercise limitation; evidence that CPET-guided dose changes improve clinical outcomes remains limited [61]. Table 3 summarizes these complementary roles.

CAC scoring quantifies calcified coronary burden and has been evaluated for cardiovascular-event prediction in a retrospective cancer cohort [62]. It can inform long-term atherosclerotic risk assessment in selected survivors but should not serve as a stand-alone exercise threshold or an acute-toxicity test.

### 5.4. Monitoring-Guided Rehabilitation Adjustment

A practical assessment sequence begins with symptoms, treatment exposure, cardiovascular risk factors, ventricular function, indicated biomarkers, and functional capacity [7,56]. Stable findings may support cautious progression, whereas new symptoms or clinically meaningful deterioration may prompt dose modification, temporary suspension, additional testing, or specialist review. A relative GLS decline greater than 15% is a clinical signal for interpretation, not an isolated exercise stop rule [7,56]. Active myocarditis, unstable arrhythmia, decompensated heart failure, or other acute toxicity requires exercise deferral until stabilization [7,16,35]. This closed-loop response sequence is the authors’ conceptual model and has not been prospectively validated.

Monitoring should answer a defined clinical question: whether exercise can start, whether a change is clinically meaningful, and whether a new symptom reflects cardiovascular injury, treatment toxicity, deconditioning, or another cause. Repeating GLS, biomarkers, CMR, or CPET without a planned management response adds burden without proven benefit. Local teams should specify who reviews results, what requires same-day contact, and how decisions reach the exercise team.

## 6. Guideline Translation and Risk-Adapted Pathways

### 6.1. Guideline Heterogeneity and Risk Stratification

ESC and American Society of Clinical Oncology (ASCO) guidance, the AHA scientific statement, the Heart Failure Association–International Cardio-Oncology Society (HFA–ICOS) tool, and the ICOS-CORE working-group statement address overlapping but different questions [7,8,18,28,63]. ESC and ASCO focus mainly on risk and surveillance; the AHA statement describes CORE components and referral considerations; HFA–ICOS structures baseline risk; and ICOS-CORE identifies evidence standards and research priorities. Table 4 makes these distinctions explicit and shows that the present review adds an evidence-labeled translation framework rather than a competing guideline.

**Figure 2:**
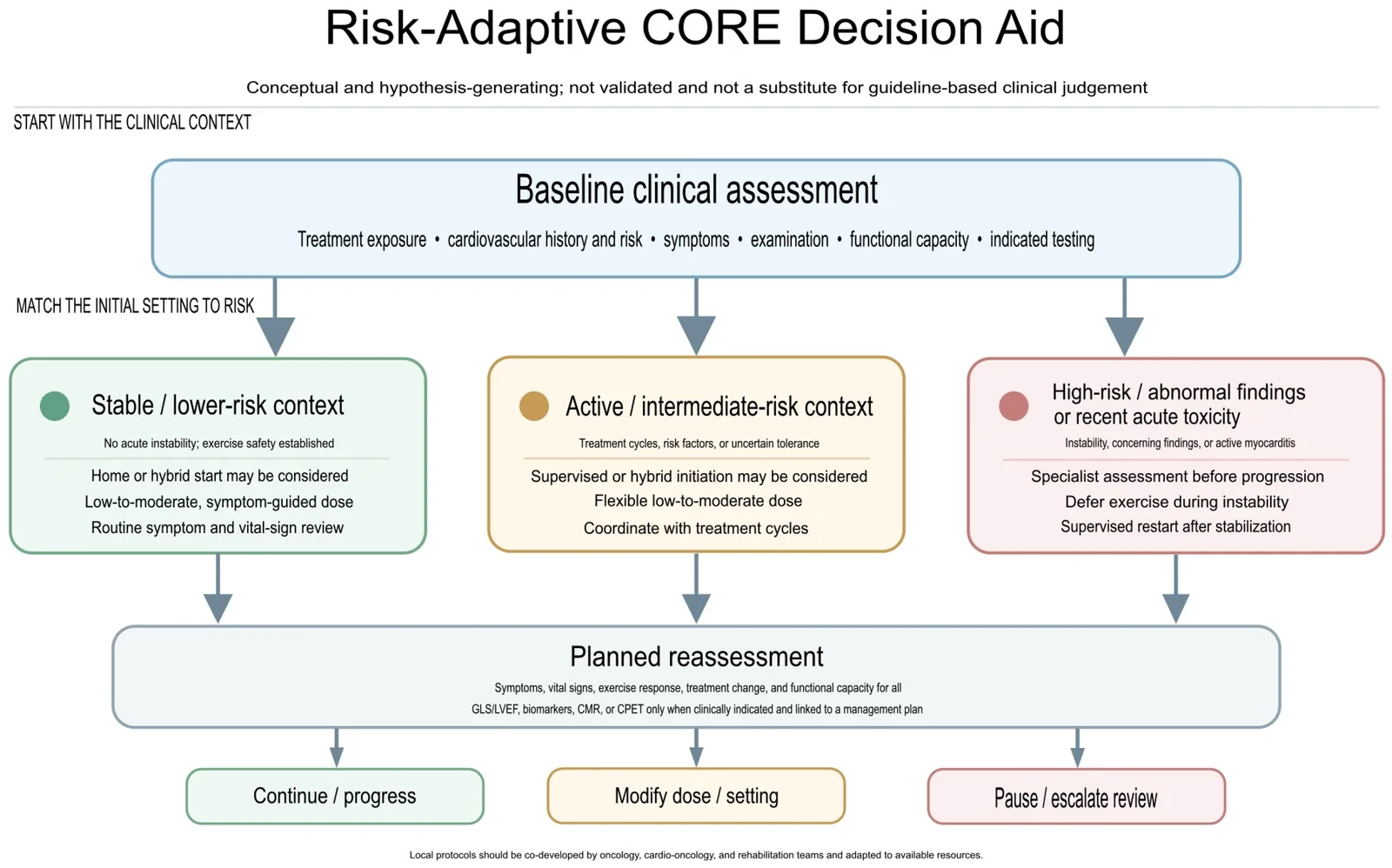
Authors’ conceptual risk-adaptive CORE decision aid. Baseline clinical assessment is linked to illustrative exercise settings, planned reassessment, and possible continuation, modification, temporary suspension, or specialist review. The pathway is hypothesis-generating, non-validated, intended for local adaptation, and does not replace guideline-based decision-making. Local protocols should be co-developed by oncology, cardio-oncology, and rehabilitation teams. CMR, cardiac magnetic resonance; CORE, cardio-oncology rehabilitation; CPET, cardiopulmonary exercise testing; GLS, global longitudinal strain; HFA–ICOS, Heart Failure Association–International Cardio-Oncology Society; LVEF, left ventricular ejection fraction.

The HFA-ICOS proforma was developed to standardize baseline cardiovascular risk assessment. A real-world breast-cancer study evaluated its association with cardiovascular events during treatment [63]. Using the proforma as an entry point for CORE is a reasonable operational proposal, but its effect on monitoring frequency, rehabilitation matching, and downstream outcomes requires validation (Figure 2).

Risk assessment must remain treatment-specific. Thoracic-radiotherapy survivors can require long-term ischemic and structural surveillance, while conventional cardiovascular risk factors require sustained prevention alongside exercise [7,41,62]. This integrated approach is clinically coherent but has not been shown to reduce cardiovascular events.

### 6.2. The Translation Gap

No major guidance document supplies a validated closed-loop CORE algorithm [7,8,18,28]. The proposed contribution of this review is to connect guideline-based risk assessment with evidence-labeled exercise, monitoring, and implementation steps while showing where the connection is supported only by feasibility data or author interpretation. This distinction is particularly important because TITAN, ONCORE, and the anthracycline exercise trial did not demonstrate consistent effects across LVEF, GLS, and biomarkers, whereas a smaller CR trial improved fitness without changing GLS or troponin [20,21,23,24].

### 6.3. A Practical Integration Pathway

The Figure 2 sequence is therefore a service-design proposal: baseline assessment informs referral and an initial setting; symptoms and treatment changes prompt routine reassessment; and clinically indicated tests may support escalation. A pathway is operational only when eligibility, starting prescription, monitoring responsibilities, response times, and return routes to oncology or cardiology are documented [64,65]. Evaluation should include reach, completion, safety, function, resource use, and equity.

At minimum, an implementable pathway should record treatment exposure, baseline risk, exercise setting, starting FITT-VP dose, monitoring triggers, the clinician responsible for abnormal findings, and escalation criteria. HFA-ICOS assessment has been used in real-world breast-cancer care, although predictive performance and downstream management effects remain under study [63]. Guideline comparison confirms broad acceptance of risk stratification but inconsistent operational detail [66]. These fields provide measurable implementation outcomes without implying that the proposed sequence is validated.

## 7. Implementation Pathways for CORE

This third core domain addresses referral and completion, delivery setting, team ownership, equity, and resource adaptation.

### 7.1. Patient-Level Barriers

Patient barriers affect referral and sustained participation. Review literature identifies intersecting financial, physical, psychological, and social barriers to cardiovascular care in women with breast cancer [26]. HEART-ACT formative work examined how a cardiac rehabilitation program could be adapted for breast-cancer survivors [67], while broader exercise-oncology work emphasizes systematic triage and referral [68]. Conventional cardiac rehabilitation is also underused by women [69]. CORE programs should therefore measure local barriers and offer flexible scheduling, remote or transport options, and individualized education.

Sex-related participation gaps require explicit measurement. Women are underrepresented in conventional cardiac rehabilitation yet overrepresented in much of the published CORE evidence because breast cancer dominates the trials [69]. Programs should report referral, enrollment, completion, safety events, and outcomes by sex, age, cancer type, socioeconomic indicator, language, race or ethnicity where appropriate, and geography, using simple dashboards or periodic audits [70]. These data can identify whether a pathway improves reach or shifts burden to patients and caregivers.

### 7.2. Provider- and System-Level Barriers

Provider and system barriers determine whether an exercise prescription is delivered. Scoping and review evidence describes fragmented service delivery and uneven integration of exercise and cardiotoxicity care [25,26]. Clinician surveys document variable knowledge and monitoring practice [27]. The ESC curriculum defines relevant professional competencies [71] but does not assign local operational ownership. Each program should specify who identifies eligible patients, reviews abnormal findings, modifies exercise, documents decisions, and finances the service.

Referral is a major implementation step. A prospective controlled study in conventional cardiac rehabilitation evaluated automatic referral and clinician-patient liaison and reported greater utilization than usual referral [72]. This coronary-disease evidence is indirect for oncology; transferability and effects on clinical outcomes require direct evaluation.

### 7.3. Delivery Models and Implementation Priorities

Hybrid and home-based delivery can address travel, mobility, and scheduling barriers. Feasibility evidence in hematologic cancer survivors [51] and a 2026 randomized trial in lymphoma survivors [50] support further evaluation of telehealth-guided exercise, while conventional cardiac telerehabilitation evidence remains indirect for cancer populations [73]. None establishes equivalence across all cardiovascular risk groups. Remote delivery should preserve symptom escalation, emergency advice, data-quality checks, and rapid access to supervised assessment. Digital devices should serve a defined clinical task rather than generate data without a response plan.

Eligibility for remote delivery should be explicit and revisited after treatment changes or new symptoms. Clinically stable patients who can monitor symptoms and use the required technology may be considered for home or hybrid sessions; unstable symptoms, recent acute toxicity, severe deconditioning, or uncertain exercise responses favor supervised assessment. Programs should also provide telephone or paper alternatives for patients without reliable devices, connectivity, privacy, or digital literacy.

Practical barriers can dilute the effectiveness of home and hybrid delivery. Unreliable connectivity or device access, limited digital literacy, privacy concerns, missing or poor-quality device data, inadequate space or equipment, competing work or caregiving demands, treatment-related fatigue, and delayed recognition of symptoms can reduce attendance, adherence, achieved intensity, and monitoring completeness [25,26,50,51,65,67,73,74]. Consequently, the delivered exercise dose may be lower or less consistent than the prescribed protocol, and comparisons with center-based care may be biased if technology-based exclusion and differential dropout are not reported. Programs should assess digital readiness, provide onboarding and technical support, use device data only when linked to a defined response, offer telephone, paper, or center-based alternatives, and track delivered dose, missing data, escalation events, and completion.

For lower-resource settings, a minimal core may comprise symptom and treatment review; blood pressure and resting heart rate; medication and cardiovascular risk assessment; a simple functional test, such as a walk or sit-to-stand test; an individualized walking and resistance plan; and a documented escalation route. Advanced imaging, biomarkers, and CPET should be reserved for clinical indications rather than treated as universal entry requirements. Task sharing with trained nurses, physiotherapists, community workers, and teleconsulting specialists is a pragmatic model that requires local safety and implementation evaluation.

Treatment-context triage remains essential. Hematologic malignancy and transplantation programs should account for cytopenias, infection risk, fatigue, and fluctuating function [51,75,76]. After ICI myocarditis or another acute cardiovascular toxicity, exercise should resume only after stabilization and specialist review [7,16,35].

## 8. Focused Research Priorities

### 8.1. Emerging Analytics: A Restricted Research Agenda

Omics, artificial intelligence (AI), automated imaging, and digital-twin models may support future phenotyping and longitudinal analysis, but current evidence is indirect, conceptual, or focused on technical performance [59,60,77,78,79]. None has demonstrated that it can safely select or adjust an exercise dose in CORE. Evaluation should proceed from external technical validation to interpretability, workflow fit, bias, safety, and prospective clinical utility before any model influences a prescription.

### 8.2. Endpoint-Focused Trials and Therapy-Specific Safety

Endpoint-focused CORE trials should prespecify treatment strata, exercise exposure, safety, adherence, functional outcomes, CTRCD, cardiovascular events, and clinically meaningful follow-up. Therapy-specific questions differ: anthracycline and anti-HER2 therapy require study of timing and cardiac-function outcomes; thoracic radiotherapy raises long-term ischemic and structural questions; and exercise after ICI myocarditis requires stabilization criteria and specialist-led safety evaluation [6,7,12,16,35,62]. Evidence from one setting should not be transferred without explicit qualification.

Research agenda: the most urgent feasible questions are (1) when exercise should begin relative to specific cardiotoxic therapies; (2) whether supervised, hybrid, and home-based delivery have comparable safety and effectiveness within defined risk strata; (3) whether monitoring-guided dose adjustment improves decisions or outcomes compared with symptom- and function-guided care; and (4) whether CORE improves treatment completion, CTRCD, cardiovascular events, equity, and cost-effectiveness.

### 8.3. Digital Delivery, Equity, and Cost-Effectiveness

Trials of digital and hybrid delivery should report access, completion, safety, staff time, technology failure, privacy, and cost, stratified by sex, age, socioeconomic indicators, language, and geography [26,50,51,65,67,74]. They should also document technology-based exclusions, access to non-digital alternatives, and whether the minimal clinical core and escalation pathway are preserved.

Overall, CORE should be treated as a testable, locally adapted care model. Direct evidence is strongest for selected functional and intermediate outcomes; pathway effectiveness and hard cardiovascular outcomes remain to be established.

Table 5 summarizes the adult clinical studies that directly anchor the main exercise efficacy, safety, and delivery conclusions, together with the principal systematic review used to contextualize this clinical evidence base. It deliberately includes positive, neutral, and null results and records cancer type, sex distribution, design, sample size, intervention, outcome, and the limited inference appropriate to each source. Guidelines, consensus statements, mechanistic studies, general background sources, and monitoring studies without a rehabilitation intervention are synthesized in their relevant narrative sections rather than treated as study-level CORE efficacy records.

## 9. Limitations of This Review

This review has several limitations. Its narrative design did not use a registered protocol, a PRISMA flow diagram, a formal risk-of-bias assessment, or a GRADE evaluation, and selection remains more vulnerable to author judgment than a systematic review. The evidence is heterogeneous across cancer types, treatment phases, cardiovascular risk, exercise doses, settings, and outcomes. Breast-cancer cohorts and women dominate the direct cardiotoxicity literature; consequently, generalizability to men, sex-diverse populations, and cancers other than breast cancer is limited. Mixed-cancer, lymphoma, prostate, and lung-cancer studies broaden the functional and delivery evidence base but do not remove this imbalance. Pediatric exercise, palliative-care rehabilitation, and detailed non-exercise supportive interventions were outside scope. Many studies are small, single-center, short-term, or focused on fitness, symptoms, risk factors, imaging, and biomarkers rather than CTRCD, cardiovascular events, or mortality. Implementation, telerehabilitation, AI, digital-twin, and precision-monitoring evidence is partly indirect or conceptual. The pathways and response rules in Figure 1 and Figure 2 and Table 2 are authors’ syntheses that require prospective validation across diverse populations, health systems, and resource settings.

## 10. Conclusions

CORE can connect cardiovascular risk assessment with exercise-centered adult cancer care. Current trials support selected fitness, functional, risk-factor, and intermediate cardiac outcomes, but they do not establish prevention of CTRCD, cardiovascular events, or mortality. Exercise should therefore be individualized to treatment exposure, symptoms, cardiovascular status, and functional capacity, with clinically indicated reassessment and specialist review when instability or toxicity is suspected.

The authors’ proposed sequence—baseline assessment, risk-adapted exercise, planned monitoring, and clinical reassessment—is a conceptual framework for local testing, not a validated algorithm. Priorities are therapy-specific timing, comparison of supervised and hybrid delivery within defined risk strata, validation of monitoring-guided dose adjustment, and trials with safety, treatment, cardiovascular, equity, and cost outcomes.

## Figures and Tables

**Table 1:** Treatment-specific cardiotoxicity mechanisms, reversibility, and monitoring markers relevant to CORE.

Therapy Class	Principal Mechanism	Reversibility	Key Monitoring Markers
Anthracyclines (e.g., doxorubicin)	Oxidative/mitochondrial injury [9,10]; cumulative dose and baseline risk guide prevention, screening, and monitoring [11]; early HF therapy may support recovery after dysfunction [15]	Cumulative injury; recovery may be incomplete [9,10]	LVEF, GLS, hs-cTn [7]
Anti-HER2 agents (e.g., trastuzumab)	Disrupted ErbB2/HER2 survival signaling [5,12]	Often reversible, but not uniformly [5,12]	LVEF, GLS, NP [7]
Thoracic radiotherapy	Dose-related ischemic risk and endothelial, vascular, and fibrotic injury [13,14]	Delayed injury; recovery may be incomplete [6,14]	Echo/GLS, CMR, or CAC as indicated [7]
Immune checkpoint inhibitors	Acute immune-mediated myocarditis requiring urgent clinical assessment [16]	Acute and potentially fulminant [16]	hs-cTn, ECG, Echo or CMR [7,16]

Note: CAC, coronary artery calcium; CMR, cardiac magnetic resonance; ECG, electrocardiography; Echo, echocardiography; GLS, global longitudinal strain; HF, heart failure; hs-cTn, high-sensitivity cardiac troponin; LVEF, left ventricular ejection fraction; NP, natriuretic peptide.

**Table 2:** Authors’ illustrative, non-validated FITT-VP starting considerations for adult CORE.

Clinical Context	Illustrative Exercise Setting and Starting Approach	Illustrative Review Considerations	Examples Prompting Clinical Reassessment
Stable/lower-risk adult survivor	Home or hybrid may be considered; low-to-moderate aerobic plus resistance; gradual volume increase [18,49].	Symptoms, blood pressure, treatment changes, and exercise response.	New chest pain, dyspnea, palpitations, syncope, or sustained intolerance.
Active therapy or intermediate-risk context	Supervised or hybrid initiation may be considered; flexible short bouts coordinated with treatment cycles.	Symptoms, vital signs, fatigue, cytopenia/infection context, and medication interactions.	Unstable symptoms, abnormal clinical findings, or deterioration during treatment.
High risk or clinically abnormal findings	Specialist-informed, supervised low-intensity start after stability is established [7,18].	Clinical review plus indicated imaging, biomarkers, or functional testing.	Pause and reassess for suspected toxicity, arrhythmia, biomarker rise, or imaging deterioration.
Recent ICI myocarditis or other acute cardiovascular toxicity	Defer during active disease; consider supervised restart only after stabilization and specialist clearance [7,16,35].	Therapy-specific clinical follow-up and a documented return-to-exercise plan.	Immediate review for recurrent symptoms or hemodynamic/electrical instability.

Note: This table is a conceptual decision aid, not a guideline, validated algorithm, or substitute for specialist assessment. Local teams should define supervision, monitoring, and escalation according to available resources and guideline-based care. FITT-VP, frequency, intensity, time, type, volume, and progression.

**Table 3:** Clinical information and limitations of complementary cardiovascular assessments relevant to CORE.

Modality	What It Detects	Key Advantages	Main Limitations
GLS (speckle-tracking echo) [56]	Subclinical change in longitudinal LV deformation	Serial, radiation-free functional measure	Vendor and image quality variability; no validated CORE dose rule
CMR [7]	Edema, fibrosis, and other tissue characteristics	Detailed tissue characterization	Cost, availability, scan time, and contraindications
hs-cTn [7]	Myocardial injury signal	Accessible serial biomarker	Nonspecific; requires timing and clinical context
BNP/NT-proBNP [7]	Hemodynamic stress or dysfunction signal	Widely available	Affected by age, renal function, and comorbidity
CPET [61]	Peak VO2 and integrated exercise response	Characterizes exercise limitation	Requires equipment and expertise; clinical outcome use is unvalidated
Coronary artery calcium score [62]	Calcified coronary atherosclerotic burden	Quantifiable long-term risk marker	Radiation exposure; not an acute toxicity test

Note: The table summarizes established clinical information provided by each test; it does not prescribe a monitoring schedule or an automatic exercise-dose rule. Testing should be clinically indicated and linked to a defined management response. BNP, B-type natriuretic peptide; CMR, cardiac magnetic resonance; CORE, cardio-oncology rehabilitation; CPET, cardiopulmonary exercise testing; GLS, global longitudinal strain; hs-cTn, high-sensitivity cardiac troponin; LV, left ventricular; NT-proBNP, N-terminal pro-B-type natriuretic peptide; VO2, oxygen uptake.

**Table 4:** Comparison of major cardio-oncology guidance and risk tools relevant to structured rehabilitation.

Guidance/Tool	Primary Purpose	Monitoring Emphasis	Relevance to This Review
ESC 2022 [7]	Treatment- and baseline-risk stratification	Risk-based imaging and biomarkers	Clinical foundation; not an operational CORE protocol
ASCO 2017 [8]	Identify higher-risk exposures and survivors	Cardiac dysfunction surveillance	Limited operational rehabilitation detail
AHA CORE statement [18]	CORE components and referral considerations	Exercise-centered multidisciplinary care	Defines scope and rationale; does not validate a closed-loop pathway
HFA–ICOS tool [63]	Structured baseline cardiovascular risk	Entry risk assessment	Potential operational input; downstream CORE effects unvalidated
ICOS–CORE 2025 [28]	Evidence standards and research priorities	Fitness, safety, and outcome reporting	Highlights heterogeneity and need for endpoint-focused trials
Current review	Evidence-labeled translation across three domains	Tests only when clinically indicated and actionable	Authors’ conceptual framework for local adaptation and prospective testing

Note: ASCO, American Society of Clinical Oncology; CORE, cardio-oncology rehabilitation; ESC, European Society of Cardiology; HFA–ICOS, Heart Failure Association–International Cardio-Oncology Society.

**Table 5:** Adult clinical studies and principal systematic synthesis directly anchoring CORE exercise and delivery conclusions.

Study/Year	Cancer/Sex	Design	N	Intervention/Exposure	Main Outcomes	Appropriate Inference
Fakhraei et al., 2022 [40]	Adult cancer survivors; predominantly women and breast cancer	Systematic review/meta-analysis	10 studies; 741	CR-based interventions.	Reporting quality and evidence were heterogeneous; CRF was the most consistent outcome.	Supports cautious synthesis; effects on cardiovascular events were not established.
Williamson et al., 2021 [19]	Mixed cancer + cardiovascular disease; 22% women	Observational cohort	442 referred; 361 completed	12-week CR.	CRF improved; program completion and higher fitness were associated with survival.	Functional evidence; survival association is non-causal.
Kirkham et al. (TITAN), 2023 [20]	Early breast cancer; all women	RCT	74 (37/37)	Up to 2 supervised moderate-intensity aerobic + resistance sessions/week (60–90 min/session) for 52 weeks; home exercise added as tolerated.	No LVEF or biomarker benefit; total and LDL cholesterol improved.	No demonstrated cardiotoxicity prevention.
Schneider et al., 2023 [23]	Breast cancer/lymphoma; 95% women	RCT	57	12 weeks: 2 supervised 90 min sessions/week + 1 home session/week. Cycling at VT1, progressing toward Borg 13 and 40 min; strength at 70–80% 1RM (2–3 × 8–12; Borg 15).	No between-group benefit for GLS, biomarkers, or VO2peak.	Neutral trial; timing and cardiac benefit remain uncertain.
Díaz-Balboa et al. (ONCORE), 2024 [21]	Early breast cancer; all women	RCT	122 (60/62)	Supervised 60 min sessions twice/week during cardiotoxic therapy (mean intervention 5.8 months): mobility/balance, strength, and 25 min cycle/treadmill exercise at 50–85% HRR (Borg 3–7/10).	No CTRCD; smaller LVEF decline; no GLS/biomarker difference; no adverse events.	Trial-specific safety and intermediate imaging evidence.
Viamonte et al., 2023 [22]	Mixed cancers/high cardiovascular risk; 77% women	RCT	80 randomized; 75 completed	8 weeks, 2 combined sessions/week. CBCR: 30–40 min cycling/walking at 50–80% HRR (Borg 12–16) + 10–15 min resistance at 40–60% 1RM; compared with CBET.	Greater VO2peak and selected risk-factor/quality-of-life improvements.	Supports supervised functional benefit, not event reduction.
Scott et al., 2021 [38]	Lung-cancer survivors; 66% women	Factorial RCT	90	48 supervised sessions (3/week for 16 weeks): cycle aerobic training at 55% to >95% VO2peak; resistance at 50–85% maximal strength; combined training; or stretching control.	Aerobic and combined training improved VO2peak; resistance alone did not; modality tolerability differed.	Supports cancer- and modality-specific prescription; not direct CTRCD evidence.
Filakova et al., 2023 [51]	Hematologic cancer, mainly lymphoma; 73% women among analyzed	Single-arm feasibility	15 enrolled; 11 analyzed	12 weeks, 3 home sessions/week: walking, Nordic walking, or cycling; 60–85% HRmax and RPE 11–13, with progressively prescribed duration (mean completed session 43.9 ± 11.6 min) and weekly calls.	Feasible, no serious adverse events; VO2peak increased in completers.	Feasibility signal; small uncontrolled study.
Chamradova et al., 2026 [50]	Lymphoma survivors; 66% women	RCT	80 (40/40)	12 weeks, 3 aerobic + resistance sessions/week, 30–50 min/session, at 60–85% HRmax; HBE included 3 initial supervised sessions and weekly telecoaching vs. CBE.	No between-group VO2peak difference; high adherence, no adverse events, lower provider cost at home.	Supports delivery comparison; no hard cardiovascular endpoints.
Kerrigan et al., 2023 [24]	Breast cancer (*n* = 28)/leiomyosarcoma (*n* = 1); all women	RCT	29	10 weeks: interval training 3 sessions/week at 60–90% HRR; usual-care comparator.	VO2peak improved relative to usual care; no between-group changes in hs-cTn or GLS.	Fitness benefit in a small trial; no demonstrated improvement in subclinical cardiac markers or hard endpoints.

Note: This table summarizes adult clinical studies that directly anchor the main conclusions on CORE exercise efficacy, safety, or delivery, plus the principal systematic review used to contextualize that evidence. Guidelines, consensus statements, other systematic syntheses, mechanistic studies, general background sources, and monitoring studies without a rehabilitation intervention are synthesized in the text according to their evidence role. This is not a PRISMA study inventory, formal risk-of-bias assessment, or GRADE evidence profile. 1RM, one-repetition maximum; CBE, center-based exercise; CBCR, center-based cardiac rehabilitation; CBET, community-based exercise training; CR, cardiac rehabilitation; CORE, cardio-oncology rehabilitation; CRF, cardiorespiratory fitness; CTRCD, cancer therapy-related cardiac dysfunction; GLS, global longitudinal strain; HBE, home-based exercise; HRmax, maximal heart rate; HRR, heart rate reserve; LDL, low-density lipoprotein; LVEF, left ventricular ejection fraction; RCT, randomized controlled trial; RPE, rating of perceived exertion; VT1, first ventilatory threshold; VO2peak, peak oxygen uptake.

## Data Availability

No new data were created or analyzed in this study. Data sharing is not applicable to this article.

## Abbreviations

The following abbreviations are used in this manuscript:

AHA	American Heart Association
AI	artificial intelligence
ASCO	American Society of Clinical Oncology
BNP	B-type natriuretic peptide
CAC	coronary artery calcium
CMR	cardiac magnetic resonance
CORE	cardio-oncology rehabilitation
CPET	cardiopulmonary exercise testing
CTRCD	cancer therapy-related cardiac dysfunction
CVD	cardiovascular disease
ECG	electrocardiography
ESC	European Society of Cardiology
FITT-VP	frequency, intensity, time, type, volume, and progression
GLS	global longitudinal strain
HFA–ICOS	Heart Failure Association–International Cardio-Oncology Society
hs-cTn	high-sensitivity cardiac troponin
ICI	immune checkpoint inhibitor
LV	left ventricular
LVEF	left ventricular ejection fraction
NP	natriuretic peptide
NT-proBNP	N-terminal pro-B-type natriuretic peptide
RPE	rating of perceived exertion
CR	cardiac rehabilitation
CRF	cardiorespiratory fitness
ICOS	International Cardio-Oncology Society
RCT	randomized controlled trial
VO2peak	peak oxygen uptake
